# Supplementary material for: Equipped for Sexual Stings? Male-Specific Venom Peptides in Euscorpius italicus
Source: Int J Mol Sci. 2022 Sep 20;23(19):11020. doi: 10.3390/ijms231911020 (PMC9570025; doi:10.3390/ijms231911020)
Supplement: Supplementary file 1 [file ijms-23-11020-s001.zip › Supplementary Materials.pdf]

Supplementary Material Table S1: Table of all venom compounds identified in *E. italicus* by a combined transcriptomic and proteomic approach.

Supplementary Material Figure S1: Compilation of MALDI TOF mass spectra of 20 venom samples from 10 individuals of *E. italicus* demonstrating a male specific expression of venom compounds. **(a)** Mass range from m/z 800 to 4500. **(b)** Mass range from m/z 3000 – 10,000.

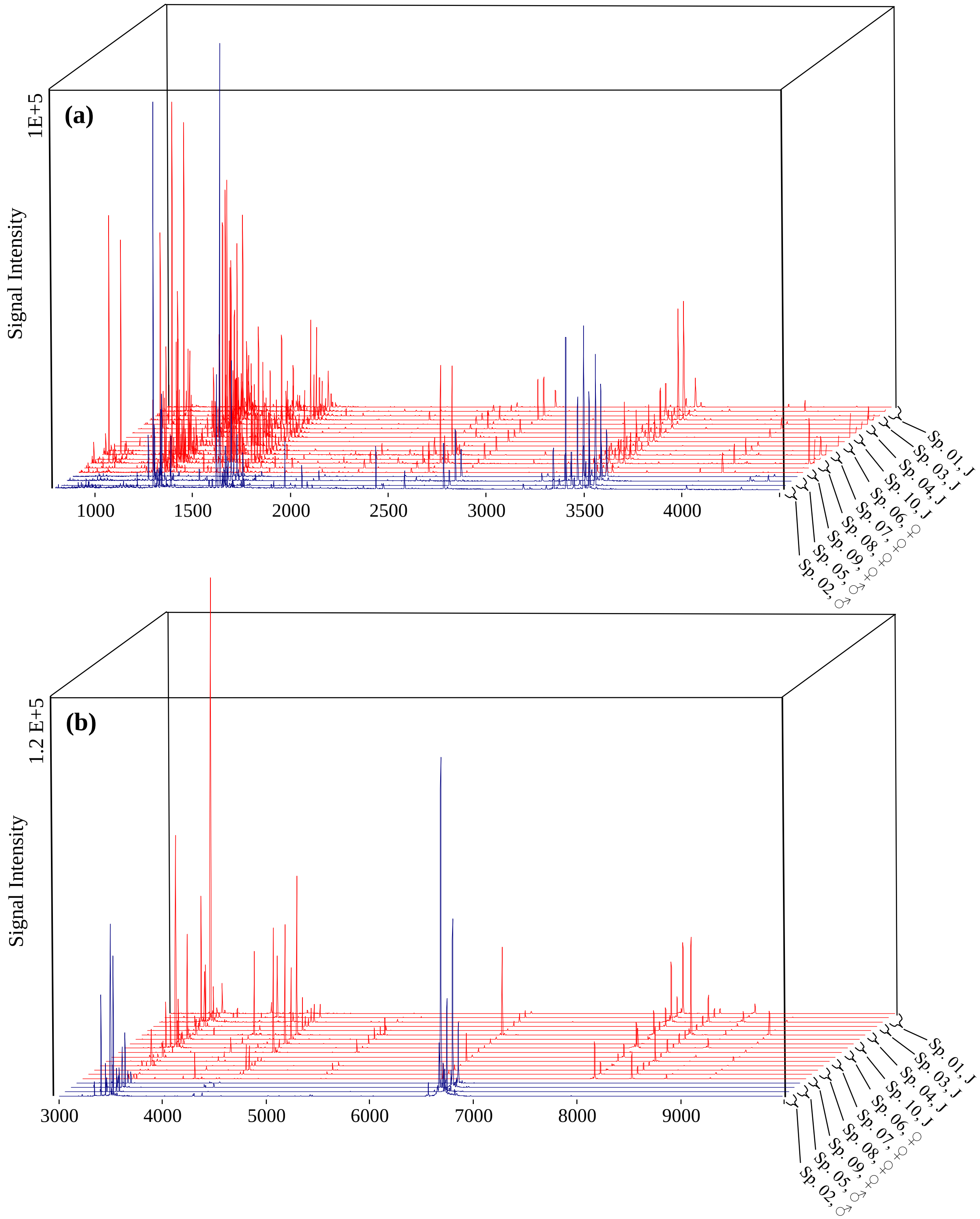

Supplementary Material Figure S2: Comparison of MALDI-TOF mass fingerprints (m/z 3000 – 10,000) of venom extracted from one specimen of *E. italicus* before (a) and after (b) transition to an adult male. Male-specific ion signals are highlighted in blue; ion signals that are missing in mass spectra of venom from males are highlighted in beige.

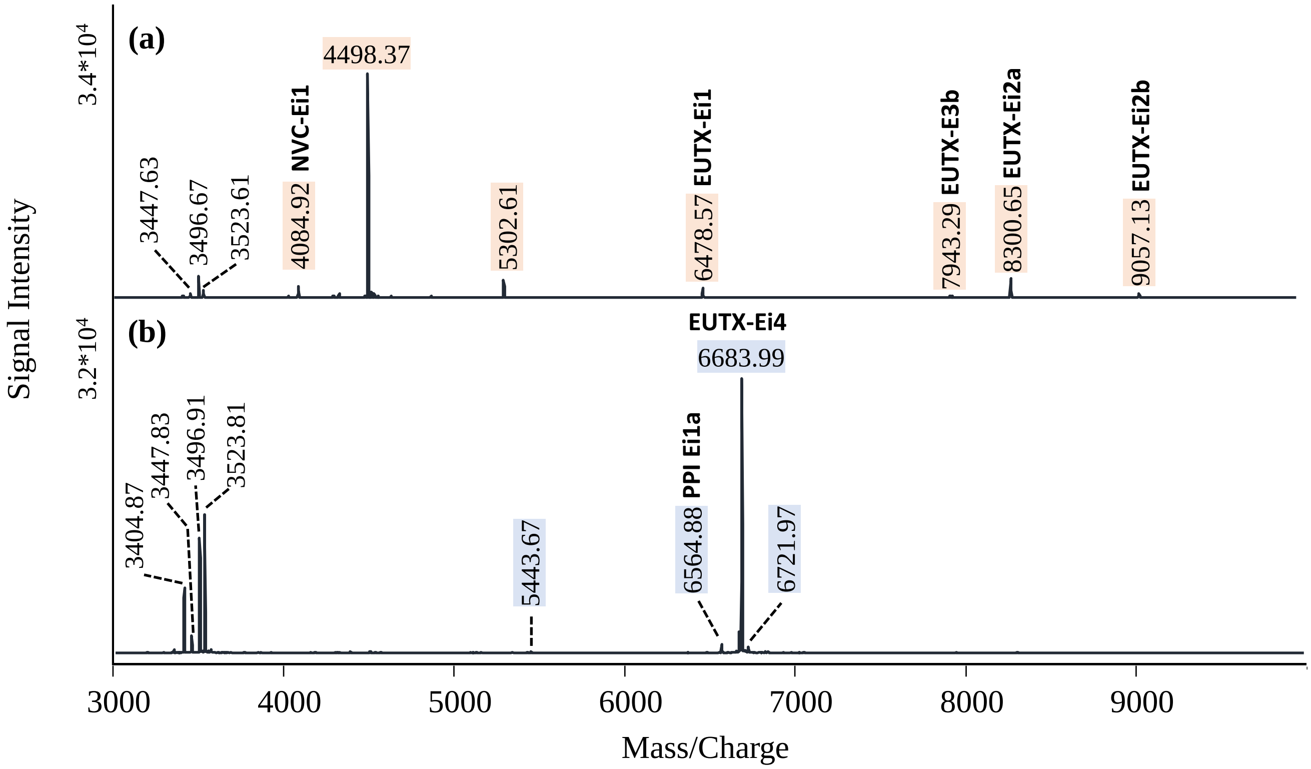

Supplementary Material Figure S3: Documentation of mating behavior for *E. italicus* (a) Promenade a deux (b) Sexual sting (c) Placement of the spermatophore (d) Close-up of the spermatophore.

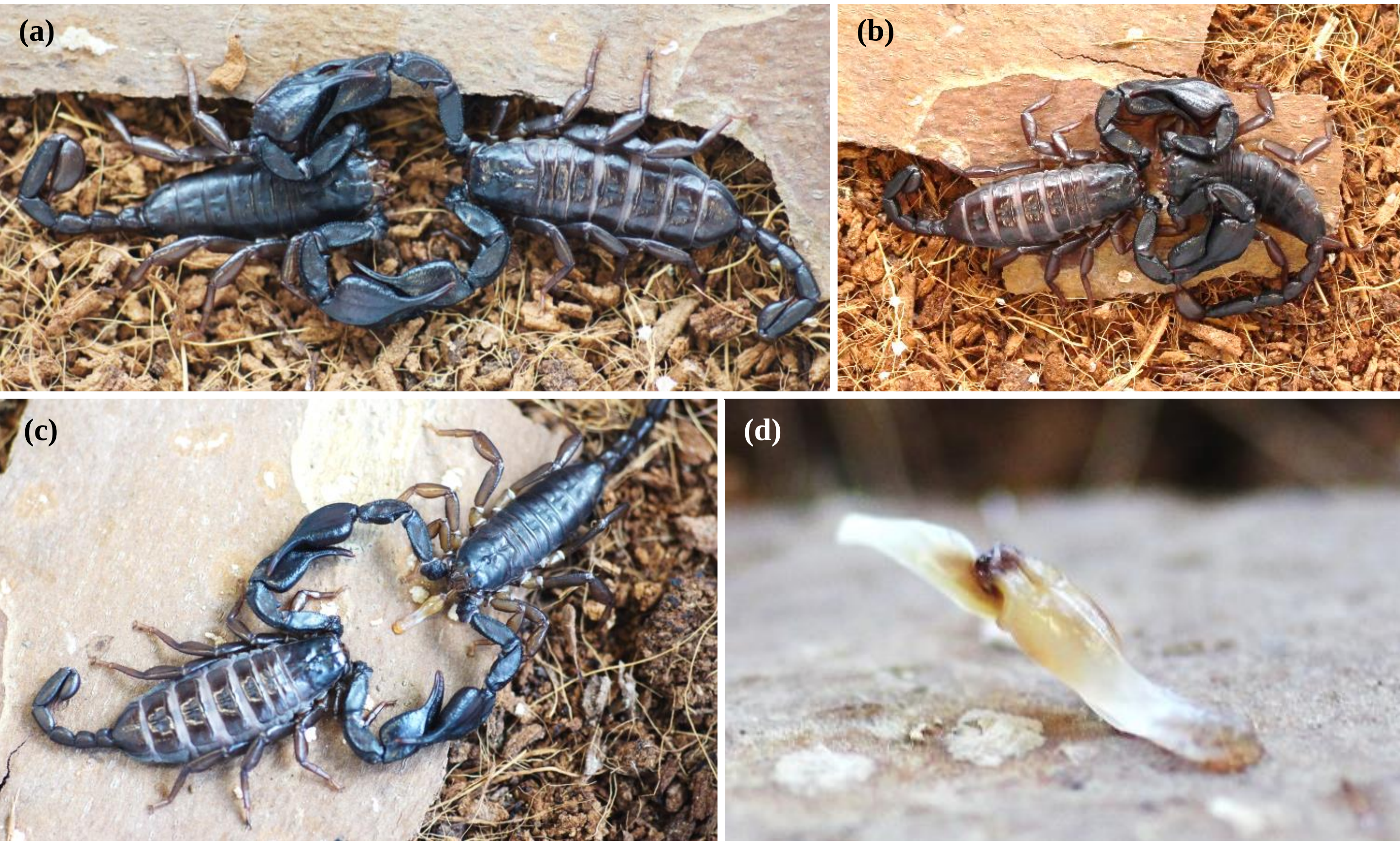

Supplementary Material Figure S4: To assess whether sex has a significant impact on the released venom amount from *E. italicus*, a linear model including specimens as fixed effects was applied, utilizing the software R (R Core Team (2022)). The venom amount was set as dependent variable with sex and maturity as explanatory variables. Venom was extracted from adult males (6 extractions; 2 specimens), adult females (12 extractions; 4 specimens), male juveniles (6 extractions; 2 specimens) and female juveniles (6 extractions; 2 specimens).

**Observations**	**30**
**Dependent variable**	Volume[µl]
**Type**	OLS linear regression

**F(10,20)**	**34.30**
**R^2**	0.94
**Adj. R^2**	0.92

	Est.	S.E.	t val.	p
**Sex**	0.50	0.24	2.12	0.05
**Maturity**	0.67	0.24	2.83	0.01
**No. of groups: Specimen**	10			
**Standard errors**	OLS			

References

R Core Team (2022). R: A language and environment for statistical computing. R Foundation for Statistical Computing, Vienna, Austria.URL https://www.R-project.org/.
